# Characteristics of the Gut Microbiota in Different Segments of the Gastrointestinal Tract of Big-Eyed Bamboo Snake (*Pseudoxenodon macrops*)

**DOI:** 10.3390/ani15203035

**Published:** 2025-10-19

**Authors:** Ruijia Xiang, Ji Chen, Ji Wang, Huina Song, Jiuyan Jiang, Fei Wu, Jingxue Luo, Mingwen Duan, Guangxiang Zhu

**Affiliations:** College of Life Science, Sichuan Agricultural University, Ya’an 625014, China; m15608167291@163.com (R.X.); 17780812758@163.com (J.C.); wangji2514@163.com (J.W.); songhuina0527@163.com (H.S.); 18227286683@163.com (J.J.); wufwf2000@163.com (F.W.); ljx12220226@163.com (J.L.); dmw0704@163.com (M.D.)

**Keywords:** big-eyed bamboo snake, gut microbiota, metagenomics, functional analysis

## Abstract

**Simple Summary:**

The gut microbiota of animals is a complex micro-ecosystem that plays a crucial role in the physiological processes of the host, including metabolism, immunity, growth and development, behavioral regulation, and disease progression. Meanwhile, its activities and composition are influenced by genetic background, diet, sample sources, and the health status of the host. However, the studies, either inside or outside of China, on the gut microbiota of vertebrates mainly focus on mammals, and less than 10% of the studies are conducted on non-mammalian animals. In this study, we analyzed the microbial composition and functional prediction across different intestinal segments of the Big-eyed Bamboo Snake (*Pseudoxenodon macrops*). We found that the small intestine contains a unique community structure, and different intestinal segments exhibit distinct metabolic functions. Our findings provide insights into the comprehensive understanding of evolution and ecology of snakes, as well as on the development of the conservative strategies for these animals.

**Abstract:**

Snakes are model animals to study energy balance, but studies on the gut microbiota of the animals are rather scarce. To fill the gap, we used metagenome sequencing to investigate the microbial community composition and adaptability in the stomach, small intestine, and large intestine of Big-eyed Bamboo Snake. The results showed that there was no significant differences in α-diversity among different gastrointestinal segments. Pseudomonadota, Bacteroidota, and Bacillota were the most abundant phyla. The dominant genera in the stomach and small intestine were similar, while those in the large intestine were distinct. The abundance of *Bacteroides, Citrobacter* and *Clostridium* was significantly higher in the large intestine than in the small intestine. The LEfSe analysis revealed that the small intestine had the most characteristic bacteria, with a total of 20 species, while the stomach and large intestine each had two species. Additionally, in the current study, we also focused on the impact of the microbial community structure on functions through functional annotations in the KEGG and CAZy. There were significant differences in the KEGG level 2 between the stomach and the small intestine. The LEfSe analysis revealed the differences in the CAZy level 2 between the large intestine and the small intestine. Overall, our study provided a comparative and contrastive analysis of the gut microbiota in different gastrointestinal segments of Big-eyed Bamboo Snake, offering valuable insights for the co-evolution mechanism of the host and the gut microbiota.

## 1. Introduction

The gut microbiota maintains a symbiotic relationship with the host, exhibiting both probiotic effects and potentially harboring certain pathogenic properties. An imbalance in the microbial community can lead to irregularities, metabolic disorders, and some irritable bowel syndrome (IBS), making it a key factor in maintaining the host`s health [1,2]. The intestinal microbiota of reptiles plays a vital role in the host’s nutritional metabolism and environmental adaptation [3]. For example, oral administration of engineered butyrate-producing symbionts in mice achieves gut–brain neural regulation, demonstrating that gut-derived butyrate can modulate brain region activity and alleviate depression-like behavior through specific gut–brain signaling pathways [4]. Additionally, factors such as the host’s genetic background and dietary preferences can jointly shape the gut microbiota, affecting its community stability and structure [3,5]. The interaction between a reptile’s immune system and the intestinal microbiota can promote the colonization of beneficial bacteria [6], and the bidirectional regulation between them is crucial for the ecological adaptation of reptiles [7].

Snakes have a wide distribution range, can adapt to various habitats, and have high species diversity. They are an important part of the Earth’s biodiversity and also serve as hosts for various bacterial pathogens [8,9,10]. Among vertebrates, snakes have an extremely strong fasting ability, which can last up to one year. After fasting, they can consume a prey equivalent to 20–40% of their body weight at one time, and convert approximately two-thirds of the intake into body tissues, demonstrating an extremely strong metabolic adaptability [11]. The digestion process of snakes may rely on the specific role of gut microbes. Therefore, studying their microbiome helps understand the energy conversion and metabolic balance mechanisms of vertebrates. However, the studies on gut microbiome in vertebrates have mainly focused on humans and other mammals, and studies on the microbiota of non-mammalian vertebrates are relatively scarce [3,12]. Among the studies related to snakes, most are conducted on captive snakes from zoos and farms, while studies on wild populations are relatively scarce [13]. To understand the diversity and composition of the intestinal microbiota of snakes, more studies among different species are needed.

Big-eyed Bamboo Snake belongs to the family Colubridae. It is distributed in the mountainous areas of Southwestern China and South Asia. It usually inhabits the crevices of rocks by streams and the shrublands. The species is nocturnal, mainly preying on small amphibians and fish [14]. The complete mitochondrial genome is approximately 19 kb in length, and the gene sequence is highly conserved, providing core data for phylogenetic studies [15]. In recent years, the high-throughput sequencing technology has been applied to construct the genome draft of this species, laying an unprecedented foundation for snake phylogenetics and comparative genomics studies [16].

In this study, we used metagenome sequencing to analyze the composition and functions of the gut microbiome of Big-eyed Bamboo Snake. The study is expected to provide important theoretical support for the currently scarce studies on snake gut microbiome and wildlife protection.

## 2. Materials and Methods

### 2.1. Sample Collection

Three adult wild male Big-eyed Bamboo Snake individuals were collected in Longcanggou National Park, Sichuan, China, at an altitude of 1427 m, on 9 May 2024. We transported the specimens into plastic bottles and transported them to the Zoology Laboratory of Sichuan Agricultural University (Approval NO.20210071). The animal care and experimental procedures were in accordance with the institutional guidelines for laboratory animals established by the Animal Care and Use Committee of Sichuan Agricultural University. Before sampling, the abdomens of the snakes were touched by hand to confirm that there was no remaining prey. Anesthesia was administered using 5% isoflurane, followed by euthanasia via injection of pentobarbital (100 mg kg^−1^). After that, aseptic dissections were performed to collect contents from the large intestines, small intestines and stomachs [17,18]. The intestinal contents were put into a sterile vial. The three snakes were designated as PM1, PM2, and PM3 (“PM” from *Pseudoxenodon macrops*). From each snake, we collected three intestinal segment samples labeled W, D, and X, respectively: W stands for “Wei” (the Chinese language for stomach); D for “Da chang” (the Chinese language for large intestine); and X for “Xiao chang” (the Chinese language for small intestine). After completing the naming and sampling process, samples were immediately frozen in liquid nitrogen and stored at −80 °C.

### 2.2. Total DNA Extraction and Library Construction

DNA was extracted using the hexadecyltrimethylammonium bromide method (CTAB). The DNA purity was detected by 1% agarose gel electrophoresis, and the concentration and purity were quantified using NanoDrop 3300 (Thermo Scientific, Shanghai, China). The final sequencing and partial data analysis were completed by Novogene Co., Ltd. (Novogene, Beijing, China). Further, 1 μg of genomic DNA from the samples was randomly fragmented into approximately 350 bp using a Covaris ultrasonic disintegrator before library construction. The library was prepared through steps such as end repair, addition of A tail, addition of sequencing adapters, purification, and Polymerase Chain Reaction (PCR) amplification. After library construction, the integrity and insert size of the library fragments were detected using AATI, and if they met the expectations, the effective concentration of the library was accurately quantified using the Quantitative Polymerase Chain Reaction (Q-PCR) (effective library concentration > 3 nM) to ensure library quality. After the library was qualified, different libraries were pooled according to the effective concentration and target output data volume requirements for PE150 sequencing.

### 2.3. Sequencing Data Processing and Species Annotation

Data analysis begins with the raw data obtained from the sequencing. Firstly, the raw reads were preprocessed using the fastp (version: 0.23.1) software. Secondly, the raw reads were then compared with the host sequence of Big-eyed Bamboo Snake using Bowtie2, to remove any contamination that may come from the host. The resulting clean reads were obtained, and assembly analysis was performed using the MEGAHIT software (version 1.2.9). Thirdly, the scaffolds obtained after assembly were interrupted at the N junctions to obtain scaffold sequences without N junctions. MetaGeneMark was used for ORF prediction, and the results were further reduced redundantly using the CD-HIT software (version 4.8.1). Bowtie2 was used to align the clean data of each sample to the initial gene catalog, and low-quality genes (reads ≤ 2) in each sample were filtered out to obtain the final gene catalog (unigenes) for subsequent analysis. At last, based on the number of reads aligned and the length of the genes, the abundance information of each gene in each sample was calculated. The Unigenes were compared with the bacterial (Bacteria) sequences extracted from the microNR database on NCBI using the DIAMOND software (v 2.1.10). Based on the LCA annotation results and the gene abundance table, the gene abundance of each classification level (phylum, class, order, family, genus, species) in each sample was obtained.

### 2.4. Diversity Analysis and Functional Prediction

Starting from the abundance tables for each classification level, we developed an abundance clustering heat map and Venn diagrams of unigenes in different gastrointestinal segments. The number of unigenes annotated at the species level was used to calculate the α diversity index. The Kruskal–Wallis test was used to determine whether the differences between groups were significant, and PCA analysis (using the R ade4 package) was performed, the MetaGenomeSeq method was used to detect the differences between groups, and LEfSe analysis was used to find the differentially expressed species between groups (with the LDA Score default set to 3). We used the DIAMOND software to compare the unigenes with the functional databases, which included the KEGG and the CAZy. For each sequence, the compared result with the highest score was selected for further analysis.

## 3. Results

### 3.1. Sequencing of the Microbiota in the Gastrointestinal Tract of Big-Eyed Bamboo Snake

A total of 9 sets of intestinal sequencing data for Big-eyed Bamboo Snake were obtained in this study (Table S2). The original data ranged from 6.17 GB to 7.74 GB, with an average of 6.80 ± 0.16GB; the data after quality control ranged from 5.98 to 7.60 GB, with an average of 6.64 ± 0.16GB; the proportion of high-quality bases (with quality values ≥ Q20) in the quality-controlled data was 97.48–98.03%, with an average of 97.65% ± 0.05%; the proportion of high-quality bases with quality values ≥ Q30 was 93.68–94.72%, with an average of 94.02% ± 0.10%. The GC content of each sample ranged from 40.657% to 44.87%, with an average of 41.34% ± 0.45%; the efficiency (the proportion of valid reads in clean reads) was 95.09–98.23%, with an average of 97.65% ± 0.33%.

**Figure 1:**
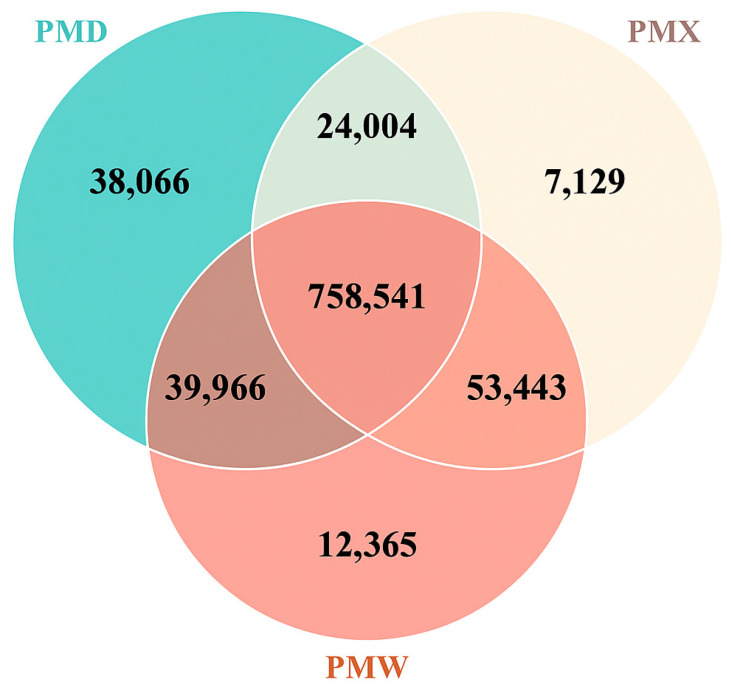
Venn diagram of differentially expressed genes in different gastrointestinal segments. PMW—Stomach; PMX—Small intestine; PMD—Large intestine.

The Venn diagram results (Figure 1) showed that the total number of common unigenes among the three gastrointestinal segments was 758,541. The large intestine had the most unique unigenes: 39,066, while the small intestine had the fewest unique unigenes: 7129. The number of common unigenes shared by the small intestine and the stomach was the highest, indicating a higher similarity between the small intestine and the stomach.

### 3.2. Diversity of Microbial Composition in Different Gastrointestinal Segments

**Figure 2:**
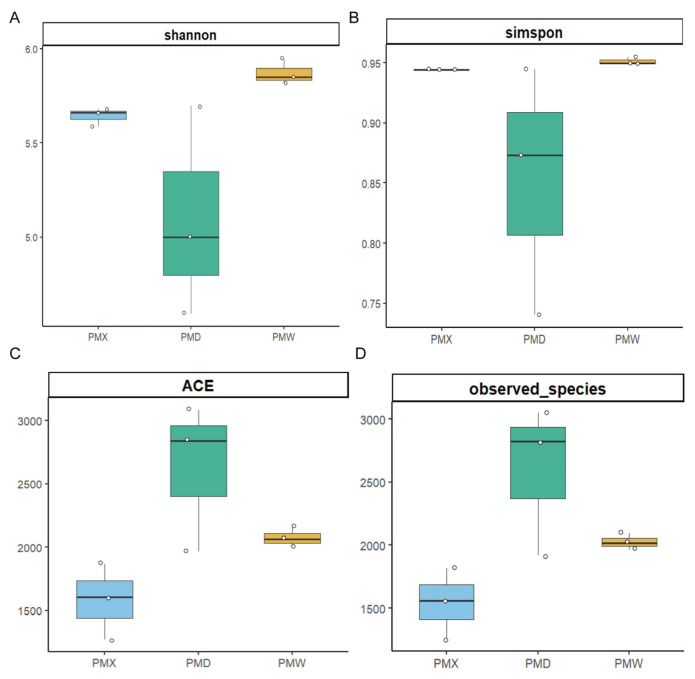
Analysis of α diversity. (**A**) Shannon. (**B**) Simpson. (**C**) ACE. (**D**) Number of observed species. PMW—Stomach; PMX—Small intestine; PMD—Large intestine.

**Figure 3:**
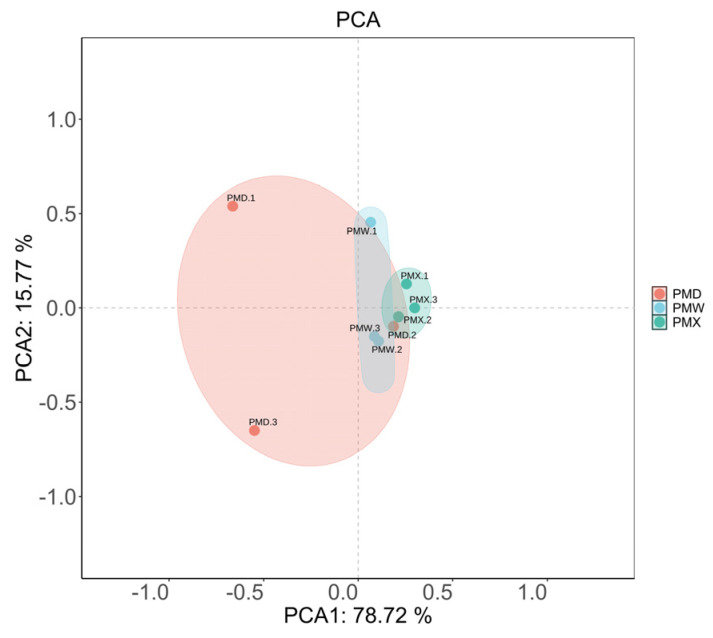
PCA analysis of different gastrointestinal segments. PMW—stomach; PMX—small intestine; PMD—large intestine.

The α-diversity analysis results (Figure 2, Table S3) showed that the goods-coverage results were all larger than 99.99%, indicating high sequencing coverage and the ability to conduct the optimal assessment of bacterial diversity. There were no significant differences in α-diversity among different gastrointestinal segments (Kruskal–Wallis test, *p* > 0.05, Table S3). The β-diversity was evaluated through PCA (Figure 3). The PCA results showed that the microbial community in different gastrointestinal segments could not be separated. The first principal component (PC1) explained 78.72% of the community structure differences, and the second principal component (PC2) explained 15.77% of the community structure differences.

### 3.3. Comparative Analysis of Microbial Composition and Structure in Different Gastrointestinal Segments

**Figure 4:**
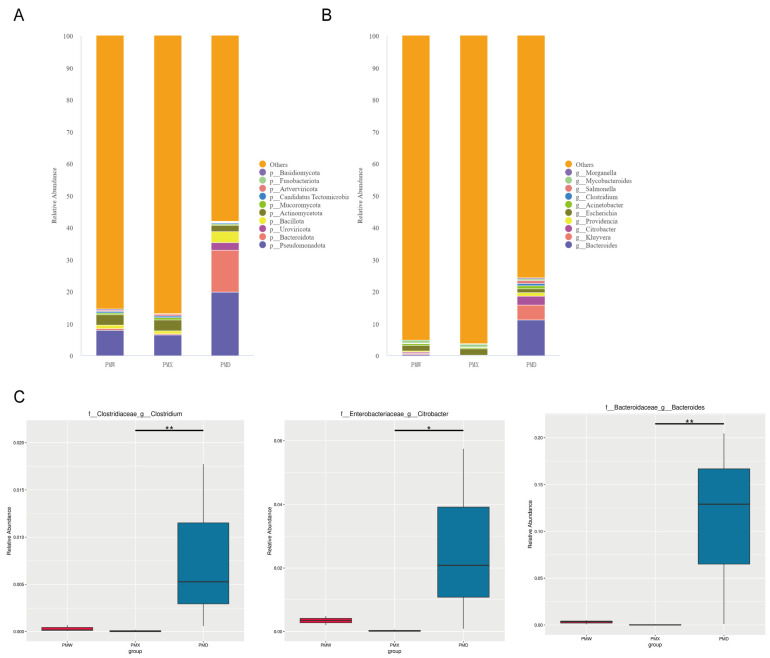
Comparative analysis of community structures in different gastrointestinal segments. (**A**) Community barplot at phylum level. (**B**) Community barplot at genus level. (**C**) Metagenomeseq analysis at genus level (*: *p* < 0.05; **: *p* < 0.01). Abbreviations: PMW—Stomach; PMX—Small intestine; PMD—Large intestine.

At the phylum level, the three most abundant phyla in the stomach were Pseudomonadota (7.88% ± 0.40%), Actinomycetota (3.21% ± 0.09%), and Bacillota (1.07% ± 0.04%). The three most abundant phyla in the small intestine were Pseudomonadota (6.52% ± 0.09%), Actinomycetota (3.34% ± 0.09%), and Bacillota (1.07% ± 0.04%). The three most abundant phyla in the large intestine were Pseudomonadota (19.90% ± 10.30%), Bacteroidota (13.04% ± 6.84%), and Bacillota (3.40% ± 1.16%). The dominant bacterial communities in the stomach and small intestine were similar, while the abundance of Bacteroidota was higher in the large intestine than in the stomach and small intestine (Figure 4A).

At the genus level, the two most abundant genera in the stomach were *Escherichia* (1.89% ± 0.05%) and *Mycobacteroides* (0.95% ± 0.03%). The two most abundant genera in the small intestine were *Escherichia* (1.97% ± 0.03%) and *Mycobacteroides* (0.99 ± 0.01%). The three most abundant genera in the large intestine were *Bacteroides* (11.2% ± 5.94%), *Kluyvera* (4.74% ± 4.49%), and *Citrobacter* (2.63% ± 1.66%) (Figure 4B).

The MetaGenomeSeq analysis revealed that there were no significant differences at the phylum level among different gastrointestinal segments. At the genus level, *Bacteroides* showed extremely significant differences in the large intestine and the small intestine (*p* < 0.01), *Citrobacter* showed significant differences in the large intestine and the small intestine (*p* < 0.05), and *Clostridium* showed significant differences in the large intestine and the small intestine (*p* < 0.05) (Figure 4C).

**Figure 5:**
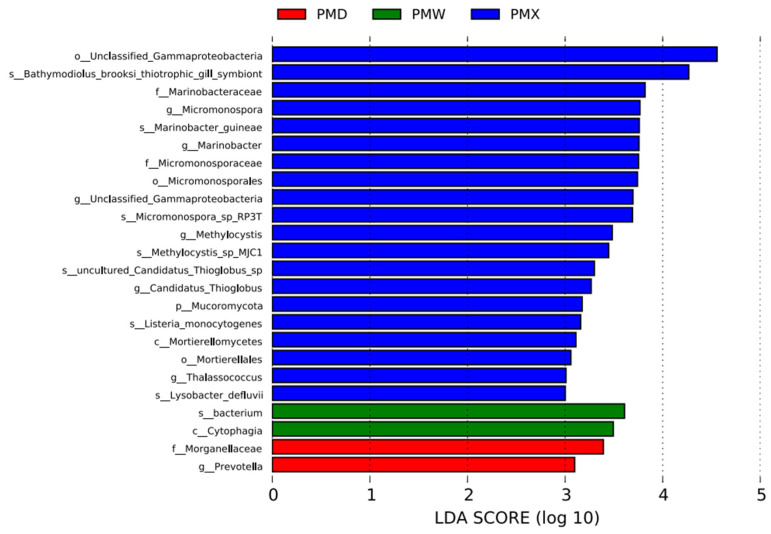
Distribution map of LDA values for different species of intestinal microbiota in various gastrointestinal segments (LDA > 3). Abbreviations: PMW-Stomach; PMX-Small intestine; PMD-Large intestine.

The LEfSe analysis suggested significant differences in the characteristic bacterial communities of different gastrointestinal segments (LDA > 3). The small intestine had the most representative bacteria, with 20 species, among which the top two bacterial communities with the highest scores were o_Unclassified_Gammaproteobacteria and S_Bathmodiolus_brooksi_thiotrophic_gill_symbiont; there were 2 species in the stomach, namely s_bacterium and c_Cytophagia; and there were 2 species in the large intestine, namely f_Morganellacese and g_*Prevotella* (Figure 5).

### 3.4. Function Prediction Analysis

**Figure 6:**
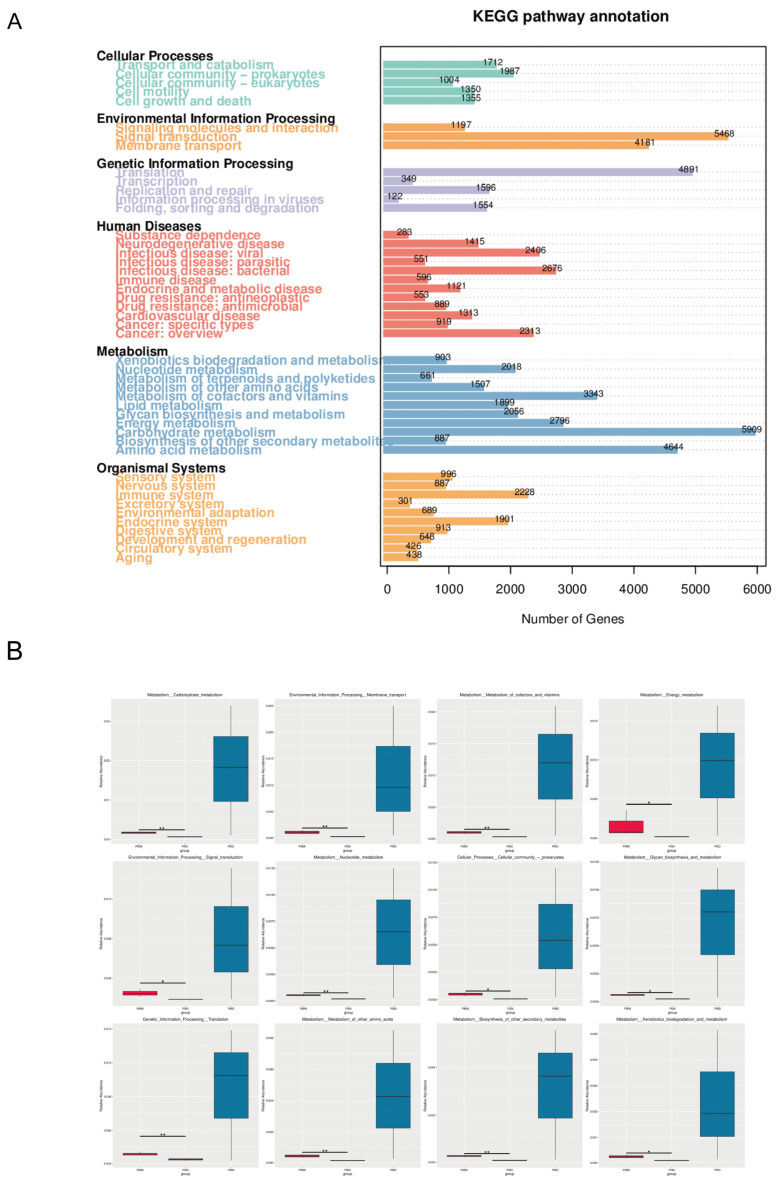
Functional analysis based on KEGG database (**A**) KEGG pathway annotated gene map (**B**) metagenomeseq analysis map (KEGG level 2) (*: *p* < 0.05; **: *p* < 0.01). Abbreviations: PMW-Stomach; PMX-Small intestine; PMD-Large intestine.

The KEGG pathway annotation results indicated that at Level 1, the majority of genes were mapped to Metabolism, presenting a significant advantage (Figure 6A). At KEGG Level 2, Carbohydrate metabolism (5909), Signal transduction (5468), Translation (4891), Amino acid metabolism (4644), and Membrane transport (4181) are the five most abundant functions of the intestinal microbiota in Big-eyed Bamboo Snake. The MetaGenomeSeq analysis revealed that among the 12 differential pathways at the KEGG Level 2, all the differences originated from the stomach and the small intestine. Among them, 8 pathways were from metabolism, 2 pathways from Environmental Information Processing, 1 pathway from Cellular Processes, and 1 pathway from Genetic Information Processing (Figure 6B).

**Figure 7:**
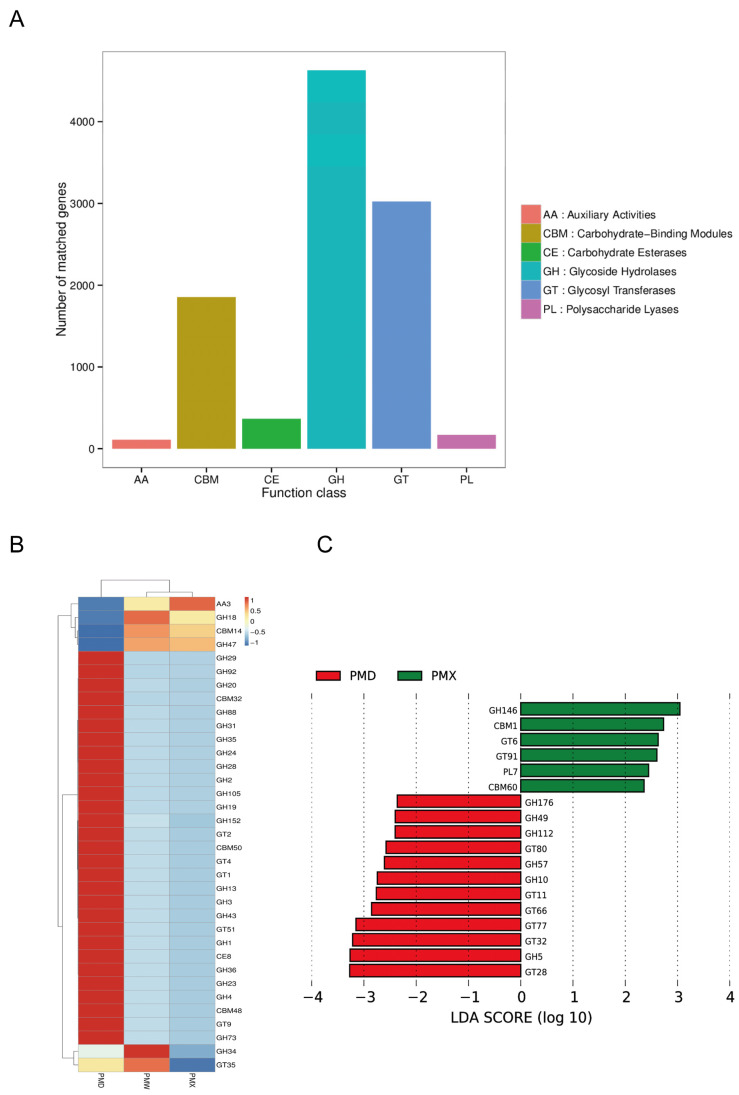
Functional analysis based on CAZy. (**A**) Functional gene annotation map of CAZy level 1. (**B**) Clustered heatmap of the relative abundance of different gastrointestinal tract microbiota in CAZymes level 2. (**C**) Distribution map of LDA values of CAZy level 2 in different gastrointestinal tract microbiota (score > 2). Abbreviations: PMW-Stomach; PMX-Small intestine; PMD-Large intestine.

After classification and annotation at Level 1, it was found that the genes of the CAZymes from the gut microbiota of snakes were related to glycoside hydrolases (GH), glycosyltransferases (GTs), and carbohydrate binding modules (CBMs), while fewer genes were related to carbohydrate esterases (CEs), accessory active enzymes (AA), and polysaccharide lyases (PL) (Figure 7A). At CAZymes Level 2, the activity of GBM14, GH18, GH47, and AA3 in the stomach and small intestine was significantly higher; the activity of GH34 and GT35 was higher in the stomach; in the large intestine, the activity of 29 enzymes was higher, and 20 of the enzymes were from the GH family, 5 from the GT family, 3 from the CBM family, and 1 from the CE family (Figure 7B). LEfSe analysis showed (Figure 7C) that there were 12 characteristic enzymes in the large intestine, mainly from the GH and GT families; there were 6 characteristic enzymes in the small intestine, mainly from the GH, GT, CBM, and PL families; no characteristic enzymes were identified in the stomach.

## 4. Discussion

The gut microbiota has a close and complex relationship with its host. The composition and structure of the microbiota can reflect the functions of the gastrointestinal tract. Microbial populations in different intestinal segments exhibit variations in abundance and distribution. A study employing absolute quantification methods and using internal standards, to measure bacterial counts in the lumen and on the mucosal surfaces of mouse cecum and colon, revealed significant spatial distribution differences across distinct intestinal segments and microenvironments. Furthermore, absolute and relative quantification results may not be consistent [19]. Metabolites produced by gut microbiota play a crucial role. For instance, hyocholic acid demonstrates potential as a novel therapeutic approach by improving key metabolic indicators of metabolic syndrome by regulating bile acid-related metabolic pathways [20]. As in other vertebrates, microbes in different parts of the digestive tract play an important role in the growth of snakes. Therefore, studying snake intestinal microbes has become an important element in snake conservation research. In this study, We used high-throughput sequencing technology to conduct a comprehensive analysis of the gastrointestinal tract of Big-eyed Bamboo Snake, revealing the community structure and function of the gut microbiota in different segments of the digestive tract.

Previous studies have shown that although the dominant phyla in the intestines of different snake species vary in composition, they generally show a trend dominated by Pseudomonadota and Bacteroidota. For example, Pseudomonadota, Bacteroidota, Actinomycetota and Bacillota are dominant in the intestine of bamboo pitviper (*Viridovipera stejnegeri*) [21]; Pseudomonadota, Bacteroidota, Bacillota and Actinomycetota are dominant in the intestine of Red-banded Snake (*Lycodon rufozonatus*) and Rose Big-tooth Snake (*Lycodon rosozonatus*) [22]; for the five gastrointestinal segments of Red-necked Keelback (*Rhabdophis subminiatus*), Pseudomonadota, Bacillota and Bacteroidota are dominant, and Fusobacteria show a significant dynamic distribution in the lower segments [12]. In this study, we also observed that the intestine of Big-eyed Bamboo Snake is dominated by Pseudomonadota and Bacteroidota at the phylum level. It is noteworthy that these snakes exhibit a high degree of similarity in their habitats and diets. They primarily inhabit forest edges, shrublands, and wetland margins, feeding mainly on small vertebrates. Consequently, despite variations in relative abundance across different regions, their similar habitats and diets result in a highly consistent overall pattern, suggesting that the composition of the snake gut microbiota may maintain a certain similarity in evolution and ecological functions.

It is worth noticing that Pseudomonadota is a facultative anaerobic, Gram-negative bacterium that participates in the metabolism of host’s carbohydrates and amino acids, lipopolysaccharide (LPS) synthesis, maintaining the redox balance of the intestinal tract; an abnormal increase in its abundance is often a sign of intestinal inflammation and metabolic disorders, and can be used as a reflecting indicator for microbial imbalance [23,24]. *Escherichia* is of Pseudomonadota and is a common genus of warm-blooded animals, which can provide the host with vitamins derived from microorganisms [25]. The bacteria in this genus can decompose carbohydrates such as polysaccharides to provide energy for the host [26]. In the human body, intestinal ecological imbalance is often closely related to the imbalance of *Escherichia* abundance, and the abundance of *Escherichia* is significantly enriched in some patients with enteritis or inflammatory bowel disease [27]. In this study, *Escherichia* is the most abundant genus in the stomach and the large intestine, indicating that this genus may play an important role in host digestion, nutrient absorption, and maintenance of intestinal homeostasis.

In contrast, the Bacteroidetes is the second most abundant phylum in Big-eyed Bamboo Snake (4.63% ± 4.20%). *Bacteroides* within the Bacteroidetes is the most abundant genus, with the highest abundance in the large intestine. The abundance of *Bacteroides* has been reported to be dominant in various mammals [28,29,30]. In some frogs, *Bacteroides* abundance has been reported to reach 20–40% [31]. In some crocodiles, *Bacteroides* has the highest abundance, indicating the high abundance of this genus across multiple vertebrate species [32]. In Red-banded Snake, *Bacteroides* has the highest abundance in the stomach and the large intestine, and the second-highest in the small intestine [22]. *Bacteroides* can efficiently decompose complex polysaccharides and dietary fibers in the intestine, produce short-chain fatty acids for host absorption, and participate in regulating intestinal immune homeostasis and barrier function, playing an important role in host energy metabolism and immune regulation [33]. Changes in the abundance of *Bacteroides* are closely related to metabolic diseases such as obesity and diabetes, and have become an important target for intestinal microecological intervention and disease prevention [34]. Therefore, the high abundance of *Bacteroides* is expected to play an important role in maintaining the stability of the intestinal structure of Big-eyed Bamboo Snake.

This study also reveals the characteristic microbial communities in different gastrointestinal segments, reflecting the highly specialized functions of the gastrointestinal tract. In the small intestine, the dominant species were uncultured Gammaproteobacteria, Bathymodiolus brooksi methane-syntrophic bacteria, Marinobacteraceae, and Micromonosporaceae; in the stomach, the phylum Cytophagia was enriched; and in the large intestine, Prevotella and Morganellaceae were characteristic. Previous studies have shown that Gammaproteobacteria are dominant in the small intestine and cloaca of cottonmouth snake (*Agkistrodon piscivorus*) [35]. In Burmese pythons (*Python bivittatus*), Gammaproteobacteria in the small intestine significantly increased after a long-term fasting [36]. The abundance of Gammaproteobacteria significantly increases during the fasting period in Burmese pythons, and is replaced by Firmicutes after feeding. This dynamic change highlights the important role of Gammaproteobacteria in maintaining microbial stability during the fasting period in snakes [37]. The genus *Micromonosporace* can produce various antimicrobial peptides, which may play a protective role in resisting pathogen colonization [38]. The phylum Cytophagia derives from Bacteroidetes, its function currently remains unclear. We speculate it may participate in the decomposition of complex organic substances in the stomach of Big-eyed Bamboo Snake. *Prevotella* is one of the core microbiota in lizards and also dominates in some turtles [3,39]. *Prevotella* with an advantage in the large intestine can decompose cellulose and other non-starch polysaccharides to produce short-chain fatty acids (SCFAs), and can also regulate the host’s immune response. *Morganella* is abundant in the intestines of crocodile lizards fed with a specific dietary pattern [39]. Therefore, we speculate that Morganellaceae may be related to the host’s dietary structure. These results indicate that the differences in the microenvironment of different gastrointestinal segments drive the diversity of the gut microbiota function in Big-eyed Bamboo Snake.

Functional prediction and enrichment analysis further indicate that metabolism is the most significant KEGG functional category, with the abundance of carbohydrate metabolism and amino acid metabolism being particularly prominent. This is consistent with the previous studies, which have shown that Red-banded Snake exhibits stronger amino acid metabolic capabilities [40]. In Chinese three-keeled pond turtles, a high abundance of carbohydrate metabolism and amino acid metabolism was also found [41]. We suppose that in the wild environment, food is relatively scarce and the survival pressure is high, which enables the gut microbiota of Big-eyed Bamboo Snake to optimize energy utilization efficiency, thereby facilitating the improvement of an individual’s adaptation and survival capabilities in the wild.

In the CAZy annotation, various enzymes from the GH and GT families are enriched in the stomach and small intestine. Previous studies on Red-banded Snake reveal that GH and GT accounted for approximately 45.7% and 37.3% of the total CAZy annotation, respectively. Among them, the GH and GT families are significantly enriched in the intestines of some crocodiles [42]. The enrichment of the GH family (such as GH18 and GH47) in the stomach and small intestine may correspond to the activity of polysaccharide-degrading enzymes, playing an important role in energy metabolism and host interaction. The diversity of the GH family in the large intestine may reflect its ability to degrade complex animal polysaccharides [43], suggesting that microorganisms can assist the host in expanding the dietary ecological niche [44]. The GT family is widely involved in the synthesis of polysaccharide substances, which helps maintain the integrity and functionality of the gastric mucosa [45]. Snakes adapt actively to the alternating states of long-term fasting and short-term overfeeding, and the high abundance of GH and GT can flexibly regulate the synthesis of carbohydrate metabolic products, reflecting the change in energy requirements, and demonstrating the importance of metabolism in different gastrointestinal tracts, especially in the stomach and intestine.

Due to the unpredictable movements and difficulty in capturing wild snakes, the sample size in this study is small (n = 3), which may limit the generalizability of the results. Although small sample sizes have been reported in previous studies [12,21,35]. Furthermore, the functional conclusions drawn in this study primarily rely on functional predictions derived from database annotations. These methods reflect functional potential rather than actual gene content or expression activity, and are subject to annotation bias and resolution limitations. Future studies should increase sample sizes to enhance the generalizability of conclusions. Additionally, multiomics approaches, including metagenomics, transcriptomics, and non-targeted metabolomics, can be integrated to establish a comprehensive research framework spanning genetic potential, functional expression, and metabolite formation. Follow-up studies may also include antibiotic resistance. Long-term antibiotic abuse may cause gut microbiota imbalance and the spread of antibiotic resistance in livestock farming. A previous article indicates that bacterial quorum sensing (QS) and its inhibitors (QS inhibitors/quenchers) represent a promising research target, but systematic validation of this approach in reptiles remains insufficient [46]. Further validation studies in this area are warranted. These methods offer greater insight into gut microbial functions and ecological roles.

## 5. Conclusions

In order to understand the differences in the structure and function of different gastrointestinal segments of the Big-eyed Bamboo Snake, and to provide a reference for the research on the intestinal microbiota of reptiles, this study systematically revealed the structural and functional characteristics of the intestinal microbiota in different gastrointestinal segments of Big-eyed Bamboo Snake for the first time. Pseudomonadota and Bacteroidota are the dominant phyla, *Escherichia* and *Bacteroides* are enriched in the stomach and the large intestine, respectively, and the unique communities in different gastrointestinal segments reflect the highly specialized functions. The CAZy and KEGG functional enrichments indicate active metabolism of carbohydrates and amino acids. In summary, our findings provide a basis for understanding the complex co-evolutionary relationship between intestinal microbiota and their hosts and provide important references for a comprehensive understanding of the evolution and ecology of snakes and the formulation of protection measures.

## Data Availability

The datasets generated for this study can be found in the SRA database of the NCBI database (Accession Number: PRJNA1293921).

## Supplementary Materials

The following supporting information can be downloaded at https://www.mdpi.com/article/10.3390/ani15203035/s1, Table S1: Sampling Information. Table S2: Statistical Information Sheet on Quality Control of Metagenomic Data. Table S3: Statistical Results of Alpha Diversity.
