# Supplementary material for: Characteristics of the Gut Microbiota in Different Segments of the Gastrointestinal Tract of Big-Eyed Bamboo Snake (Pseudoxenodon macrops)
Source: Animals (Basel). 2025 Oct 19;15(20):3035. doi: 10.3390/ani15203035 (PMC12561061; doi:10.3390/ani15203035)
Supplement: Supplementary file 1 [file animals-15-03035-s001.zip › animals-3873272-supplementary.pdf]

Table S1 Sampling Information

Sample	Place	Altitude	Sampling site	Date	Latitude and longitude
PM1	Longcanggou, Yingjing County, Sichuan Province	1427m	Stomach(PMW.1), small intestine(PMX.1), large intestine Stomach(PMD.1)	2024.6.9	102.88879993°E, 962838384°NN
PM2			Stomach(PMW.2), small intestine(PMX.2), large intestine Stomach(PMD.2)		102.88879993°E, 29.62839083°N
PM3			Stomach(PMW.3), small intestine(PMX.3), large intestine Stomach(PMD.3)		102.88845792°E, 29.62805507°N

Table S2 Statistical Information Sheet on Quality Control of Metagenomic Data

SampleID	Raw_Base (G)	Clean_Base(G)	Clean_Q20(%)	Clean_Q30(%)	Clean_GC (%)	Effective(%)	NonHost_Base(G)
PMW.1	6.29	5.98	97.66	94.13	41.2	95.09	3.47
PMX.1	7.74	7.6	97.65	93.98	40.72	98.11	3.96
PMD.1	6.8	6.63	98.03	94.72	44.87	97.56	5.52
PMW.2	6.64	6.52	97.59	93.88	40.57	98.2	3.66
PMX.2	6.84	6.68	97.48	93.68	41.21	97.78	3.56
PMD.2	7.24	7.09	97.55	93.85	40.7	97.95	3.61
PMW.3	6.84	6.72	97.57	93.89	40.58	98.23	3.58
PMX.3	6.6	6.47	97.63	93.99	40.72	98.08	3.64
PMD.3	6.17	6.04	97.72	94.13	41.52	97.89	4.19

Table S3 Statistical Results of Alpha Diversity

sample	ACE	chao1	shannon	simpson	observed_species	goods_coverage
PMX.1	1606.816	1630	5.662392	0.944251	1560	0.99993585
PMX.2	1868.114	1879.084	5.677746	0.944829	1815	0.999921032
PMX.3	1270.166	1274.688	5.587557	0.944135	1262	0.999981735
PMD.1	3079.84	3082.843	4.99892	0.873036	3049	0.999982503
PMD.2	1963.58	1971.923	5.697276	0.944836	1917	0.999922755
PMD.3	2840.315	2852.015	4.592059	0.740364	2821	0.999985236
PMW.1	2063.2	2068.15	5.944098	0.954774	2018	0.999931236
PMW.2	2157.633	2159.248	5.815807	0.949247	2095	0.999916849
PMW.3	2000.269	2010.559	5.849351	0.949788	1963	0.999941058

Indicator	Chi-squared	df	p-value
ACE	5.6	2	0.06081
chao1	5.6	2	0.06081
shannon	5.6	2	0.06081
simpson	5.6	2	0.06081
